# Identification of novel autoantigens as potential biomarkers in juvenile idiopathic arthritis associated uveitis

**DOI:** 10.3389/fped.2022.1091308

**Published:** 2023-01-09

**Authors:** Sabine Arve-Butler, Anki Mossberg, Fredrik Kahn, Seyed Morteza Najibi, Elisabet Berthold, Petra Król, Bengt Månsson, Robin Kahn

**Affiliations:** ^1^ Department of Rheumatology, Clinical Sciences Lund, Lund University, Lund, Sweden; ^2^ Wallenberg Center for Molecular Medicine, Lund University, Lund, Sweden; ^3^ Department of Pediatrics, Clinical Sciences Lund, Lund University, Lund, Sweden; ^4^ Department of Infection Medicine, Clinical Sciences Lund, Lund University, Lund, Sweden

**Keywords:** autoantigen, autoantibodies, biomarker, uveitis, juvenile idiopathic arthiritis, juvenile idiopathic arthiritis associated uveitis, anti nuclear antibody (ANA)

## Abstract

**Background:**

Many children with juvenile idiopathic arthritis (JIA) have autoantibodies, targeting nuclear components (anti-nuclear antibodies, ANA). ANA in JIA is associated with uveitis, an eye inflammation which may cause permanent vision impairment if not detected and treated. However, ANA-testing is neither specific nor sensitive enough to be a clinically reliable predictor of uveitis risk, and the precise autoantigens targeted by ANA in JIA are largely unknown. If identified, specific autoantibodies highly associated with uveitis could be used as biomarkers to facilitate identification of JIA patients at risk.

**Methods:**

Antibodies from six ANA-positive, oligoarticular JIA patients, with and without uveitis, were explored by two large-scale methods: (1) screening against 42,100 peptides on an autoimmunity profiling planar array, and (2) immunoprecipitations from cell lysates with antigen identification by mass spectrometry. Three hundred thirty-five peptide antigens, selected from proteins identified in the large-scale methods and the scientific literature were investigated using a bead-based array in a cohort of 56 patients with oligoarticular- or RF-negative polyarticular JIA, eight of which were having current or previous uveitis.

**Results:**

In the planar array, reactivity was detected against 332 peptide antigens. The immunoprecipitations identified reactivity towards 131 proteins. Only two proteins were identified by both methods. In the bead-based array of selected peptide antigens, patients with uveitis had a generally higher autoreactivity, seen as higher median fluorescence intensity (MFI) across all antigens, compared to patients without uveitis. Reactivity towards 17 specific antigens was significantly higher in patients with uveitis compared to patients without uveitis. Hierarchical clustering revealed that patients with uveitis clustered together.

**Conclusion:**

This study investigated autoantigens in JIA and uveitis, by combining two exploratory methods and confirmation in a targeted array. JIA patients with current or a history of uveitis had significantly higher reactivity towards 17 autoantigens and a generally higher autoreactivity compared to JIA patients without uveitis. Hierarchical clustering suggests that a combination of certain autoantibodies, rather than reactivity towards one specific antigen, is associated with uveitis. Our analysis of autoantibodies associated with uveitis in JIA could be a starting point for identification of prognostic biomarkers useful in JIA clinical care.

## Introduction

Juvenile idiopathic arthritis (JIA) is a rheumatic disease affecting children. The JIA diagnosis covers several disease subtypes, each with a different set of clinical features. The common denominator is unexplained persistent arthritis which debuts before the age of 16 (1, 2). There are currently seven subtypes of JIA; oligoarticular, rheumatoid factor (RF) negative polyarticular, RF-positive polyarticular, juvenile psoriatic, enthesitis-related, systemic, and undifferentiated JIA (1, 2). The pathology of most JIA subtypes, except systemic JIA, is suggested to be initiated by autoimmune reactions. For instance, like many other autoimmune diseases, JIA is associated with HLA class II genetic variants and a high proportion of patients have autoantibodies (3, 4).

Approximately 40% of all JIA patients have antinuclear antibodies (ANA) (5). ANA are associated with certain JIA phenotypes and outcomes, and ANA-positivity has therefore been suggested as a classification criteria for redefined JIA subgroups (6–8). The clinical features associated with ANA are early disease onset, female gender, asymmetric arthritis, oligoarticular or RF-negative polyarticular arthritis, and an increased risk for uveitis (9–11).

Uveitis affects 10%–30% of JIA patients, and can cause permanent visual impairment if not detected and treated timely (12, 13). Today there are no biomarkers which adequately can predict the risk for this extra-articular manifestation. ANA are associated with uveitis, but as many JIA patients are positive for ANA without ever having uveitis, it has insufficient specificity. Additionally, the ANA-tests used in clinical practice today were designed for other diseases, not evaluating JIA-specific antigens. Despite many JIA patients having ANA (5, 14–16), very few have reactivity towards extractable nuclear antigens (E-ANA) in clinical assays used for identification of ANA antigens in other rheumatic diseases (17). Therefore, to detect uveitis early, children with JIA must undergo frequent ophthalmological examinations as part of their clinical care. A specific antibody test which could stratify risk of uveitis among JIA patients would thus be cost-effective, reduce unnecessary hospital visits, and help identify children at risk prior to symptom onset.

In this study, we aimed to identify specific autoantigens associated with uveitis among patients with oligoarticular- and RF-negative polyarticular JIA. The oligoarticular- and RF-negative polyarticular JIA subtypes were selected based on their shared immunological features, association with ANA, and increased risk of chronic uveitis (6, 10, 14).

## Materials and methods

### JIA patients

Patients with oligoarticular and RF-negative polyarticular JIA were eligible for study participation. JIA patients were recruited from the pediatric rheumatology clinic at Skåne University Hospital, Lund, Sweden. Serum and plasma samples were collected 1990–2003 and 2018–2021, on occasions where patients were taking routine blood samples for disease monitoring. Samples were stored at −80°C until use.

For the autoimmunity profiling planar array and immunoprecipitations, plasma samples from six patients with oligoarticular JIA were used. Equal volumes of plasma were mixed into two pools with three patients in each, grouped by uveitis. The plasma samples were collected 2018–2020. Clinical features are summarized in Table 1.

**Table 1:** Description of patients in plasma pools.

Patient	Gender	Age (years)	Diagnosis	Uveitis	ANA
1	F	11	P oligo	1	+
2	F	16,5	P oligo	1	+
3	F	5	P oligo	1	+
4	F	15	P oligo	0	+
5	F	3,5	P oligo	0	+
6	M	4,5	P oligo	0	+

P oligo, persistent oligoarticular JIA.

For the targeted array, serum samples from 56 patients with oligoarticular or RF-negative polyarticular JIA were used. Clinical features are summarized in Tables 2, 3. Samples from 17 of the 56 patient samples were collected 1990–2003, and 39 samples collected 2018–2021. All patients fulfilling the following criteria were included in the study: (1) serum sample available, (2) clinical data on uveitis, ANA, age at sample collection, and sex.

**Table 2:** Description of patient cohort included in bead array, grouped by subtype.

	Total JIA	Oligo persistent	Oligo extended	RF- poly
Patients *n* (% total)	56	35 (63)	10 (18)	11 (20)
Female *n* (%)	38 (68)	24 (69)	7 (70)	7 (64)
Age (years) Median (range)	10.25 (1.5–17)	10 (1.5–17)	9.75 (1.5–15)	11 (2–16)
ANA *n* (%)	40 (71)	25 (71)	8 (80)	7 (64)
Uveitis *n* (%)	8^a^ (14)	5 (14)	1 (10)	2 (18)

^a^ Seven patients had chronic uveitis and one had acute uveitis. Oligo, oligoarticular; poly, polyarticular.

**Table 3:** Description of patient cohort included in bead array, grouped by uveitis.

	Uveitis	No uveitis
Patients^a^ *n* (% total)	8 (14)	48 (86)
Female *n* (%)	7 (88)	31 (65)
Age (years) Median (range)	10.5 (3–16.5)	10.25 (1.5–17)
ANA *n* (%)	8 (100)	32 (67)
Oligo persistent *n* (%)	5 (63)	30 (63)
Oligo extended *n* (%)	1 (13)	9 (19)
RF- poly *n* (%)	2 (25)	9 (19)

^a^ None of the patients had received biological DMARDs before blood sampling. Oligo, oligoarticular; poly, polyarticular.

For samples collected 2018–2021, informed written consent to donate samples for the study was obtained from all patients and/or their guardians, and the study was approved by the Regional Ethical Review Board for Southern Sweden (LU2016/128). For samples collected 1990–2003, informed consent to collect and store samples was obtained from all patients and/or their guardians, and permission to use these samples for research purposes was later obtained by the Regional Ethical Review Board for Southern Sweden (LU2005/411).

### Study design

**Figure 1:**
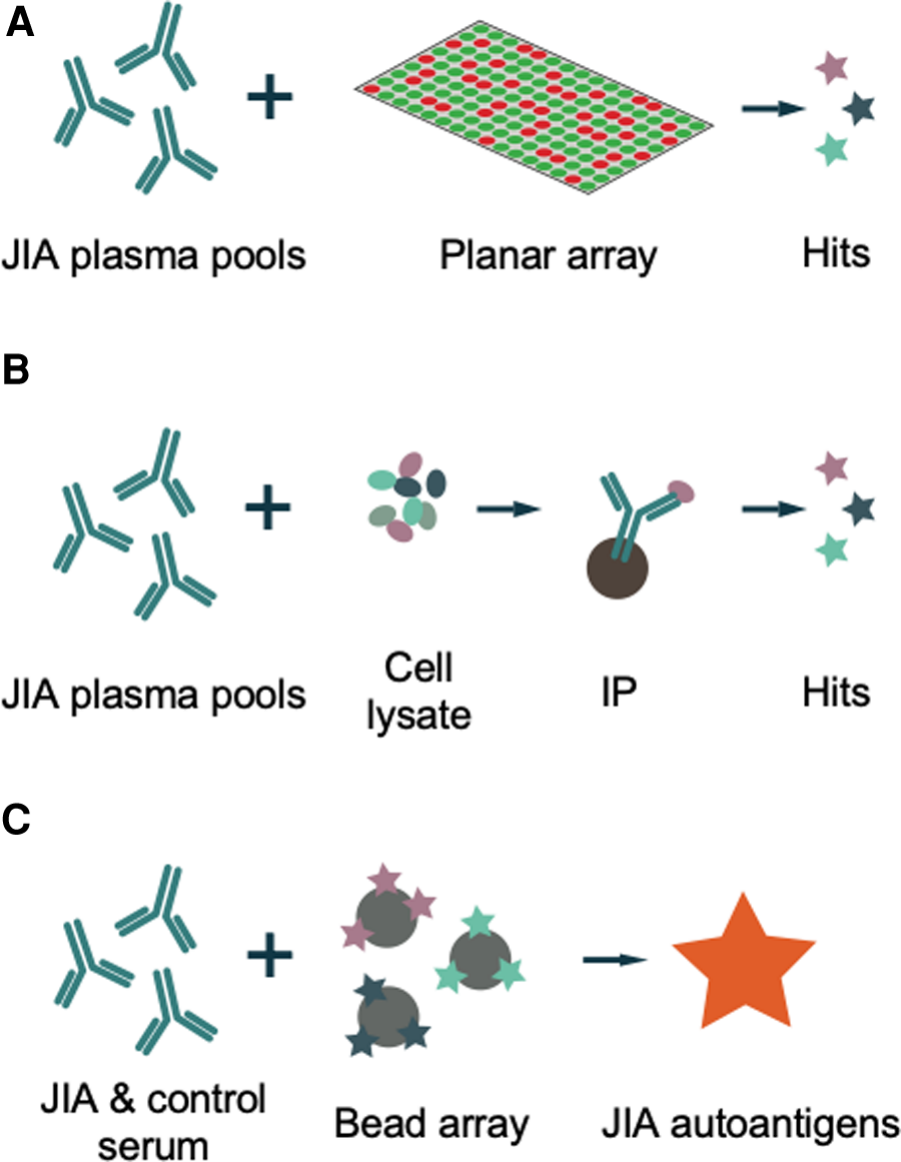
Schematic methodology. (**A**) JIA plasma is analyzed for reactivity towards 42,100 peptide antigens on a glass slide in a planar array. (**B**) JIA plasma is incubated with whole cell protein extracts, proteins captured by JIA IgG are immunoprecipitated (IP) and identified by mass spectrometry. (**C**) Hits from A and B are coupled to beads and reactivity analyzed in serum from a cohort of 56 patients with oligoarticular- or RF-negative polyarticular JIA, 8 of which with current or history of uveitis, to identify uveitis-related autoantigens.

We performed two methods of non-biased, large-scale searches for potential JIA- and uveitis-related autoantigens: A planar array and immunoprecipitations from whole cell protein extracts (Figures 1A,B). The experiments were performed using two plasma pools described above.

As a next step, PrEST antigens were selected for a targeted array based on hits from the exploratory methods, and proteins previously described as potential autoantigens in JIA and/or autoimmune eye disease. The selected PrEST antigens were investigated in a bead-based array, using a cohort of 56 JIA patients with oligoarticular- and RF-negative polyarticular JIA, with and without uveitis (Figure 1C).

PrEST antigen in the bead-based autoantibody array were chosen based on hits in the autoimmunity profiling planar array and immunoprecipitations, as well as from proteins described as autoantigens in JIA and autoimmune eye disease (18–24). All PrEST antigens with hits in both JIA plasma pools on the planar array were included in the bead array, as well as PrEST antigens from the majority of the proteins captured by both pools in the immunoprecipitations. Antigens from proteins detected by immunoprecipitation, but unlikely to be produced by the cell type used for protein extract were not included in the bead array. The remaining PrEST antigens were chosen to represent all subcellular locations and protein families identified in planar array and immunoprecipitations, together with antigens from proteins recognized as autoantigens in JIA or autoimmune uveitis in the literature (4, 18–20, 22–27).

### Autoimmunity profiling planar array

The array was performed at SciLifeLab, Stockholm. A total of 42,100 unique peptide antigens (Protein Epitope Signature Tags, PrEST antigens), representing 18,000 proteins, were generated as described in (28) together with the Human Protein Atlas project (www.proteinatlas.org) (29). PrEST antigens are peptides of 50–150 amino acids length, tagged with N-terminal His6 and albumin binding protein (His6ABP). The PrEST antigens were expressed in *E. coli* and placed in spots on glass slides.

The plasma pools were diluted 1:100 in phosphate buffered saline (PBS) with 0.1% Tween20, supplemented with 3% bovine serum albumin (BSA), 5% milk, and 160 µg/ml recombinant His6ABP and pre-incubated for 1 h. The samples were then added to the peptide array glass slides. After 1 h, the slides were washed with 0.01% PBS-Tween20 followed by incubation with 0.645 μg/ml hen-anti-His6ABP (Human Protein Atlas) for 1 h to label all peptides. Goat-anti-human IgG-AlexaFluor 647 (Invitrogen A21445) and goat-anti-chicken IgY-AlexaFluor 555 (Invitrogen, A21437) were diluted 1:15,000 and incubated on the slides for 1 h. The slides were washed with 0.01% PBS-Tween20 and scanned in a Capital Bio LuxScan at 10 μm resolution.

Images were analyzed in GenePix Pro 5.1 and statistical analysis performed in R. Median fluorescence intensity (MFI) for both peptide amount (green channel) and autoantibody presence (red channel) was measured for each peptide spot. Criteria for quality control exclusion of peptides from analysis were spot size <30 pixels, spot green MFI <10 standard deviations of the local background, or peptide not matching a valid Ensmbl Gene ID. The cut-off for binary transformation was the mean across all spots plus four times the standard deviation.

### Immunoprecipitations

Protein extracts were prepared from 3 × 10^6^ CCL-25 cells (WISH, ATCC), grown to 80% confluency in DMEM (Gibco) supplemented with 10% fetal calf serum. Cells were washed in PBS and lysed in RIPA buffer (Pierce) supplemented with protease inhibitors (cOmplete, Roche Diagnostics) at 4°C for 2 h. After centrifugation at 14,000 g for 10 min at 4°C, the supernatant was collected. The pellet was resuspended in PBS with cOmplete protease inhibitor and sonicated in 10 cycles of 30 × 1s at 60% amplitude (Hielscher, UP100H Lab Homogenizer). The sonicated pellet was centrifuged at 14,000 g and the supernatant pooled with the supernatant from the first centrifugation. Protein concentration in the cell extract was determined by BCA Protein Assay Kit (Pierce).

For the IP, 10 µl of patient plasma was added to 600 µg protein extract in PBS and incubated with end-over-end rotation at 4 °C o/n. Antibodies and bound antigens were captured by Protein G-coupled Dynabeads (Invitrogen, Thermo Fisher Scientific). Proteins were eluted with glycerin pH 2.8 and neutralized in ammonium carbonate pH 9.

### Antigen identification by mass spectrometry

Eluted proteins were prepared for liquid chromatography mass spectrometry (LC-MS) by reduction in 10 mM dithiothreitol for 30 min at 56 °C and alkylation in 20 µM iodoacetamide for 30 min at RT. Proteins were digested by trypsin (Promega, Sequencing Grade) over night at 37 °C. Trypsin was inactivated by acidification. Peptides were desalted using Ultra Microspin C18 spin columns (The Nest Group) and adjusted to 0.5 µg/µl in dH_2_O with 2% acetonitrile and 0.1% trifluoroacetic acid.

Diluted peptides, 1 µg, were loaded into a Tribrid Fusion mass spectrometer (Thermo Fischer Scientific). Peptides were concentrated on an Acclaim PepMap 100 C18 precolumn (Thermo Fisher Scientific) and separated on an Acclaim PepMap RSLC column (nanoViper) at 40°C and flow rate 300 nl/min. Peptides were eluted by a nonlinear gradient of 3% to 90% ACN in dH_2_O with 0.1% formic acid for 80 min. The Orbitrap Fusion was operated in the positive data-dependent acquisition (DDA) mode. Full MS survey scans from m/z 350–1,350 with a resolution of 120,000 were performed in the Orbitrap detector.

The raw DDA data were analyzed with Proteome Discoverer™ 2.5 software (Thermo Fisher Scientific). Peptides were identified using SEQUEST HT against UniProtKB human database (UP000005640_9606). Proteins detected with FDR confidence value ≥95% taken into consideration for the subsequent peptide selection. Proteins selected for further analysis is shown in Supplementary Table S2.

### Bead-based autoantibody array

The array was performed at SciLifeLab, Stockholm. Selected peptides were coupled to color-coded magnetic beads (MagPlex, Luminex Corp, Austin TX) by COOH-NH_2_ binding. Assay controls were bare beads, and beads coupled to His6ABP, Epstein-Barr nuclear antigen 1 (EBNA1) and rabbit-anti-human IgG (Jackson Immunoresearch). Coupling efficiency was analyzed using hen-anti-His6ABP antibodies (Human Protein Atlas).

Serum samples were diluted 1:250 in 0.05% PBS-Tween20, supplemented with 3% BSA, 5% milk, 160 µg/ml recombinant His6ABP, and incubated for 1 h prior to incubation with antigen-coated beads for 2 h. Antigen-bound antibodies were fixated using 0.02% paraformaldehyde for 10 min and stained with goat-anti-human IgG Fc-PE (eBioscience, 12-4998-82) for 30 min. Samples were analyzed in a FlexMap 3D instrument (Luminex Corp, Austin TX).

### Data processing to identify relevant JIA autoantigens in the bead array

Data processing was performed in R. Quality control analyzed both MFI and bead counts. Four antigens (HPRA007628 (EYA2), HPRA009102 (GFAP), HPRA011087 (PKM), and HPRA012741 (ARG1)) were excluded based on MFI below (MFI(empty bead) + 3*standard deviation(empty bead)).

Raw MFI values were log2 transformed and analyzed separately for each antigen. Age, anti-human IgG, and His6ABP were considered covariates in a model selection procedure. The models were t-test (reduced model without covariates), only one covariate included, or all covariates included (full model: main effects without interaction). The best model was selected by the lowest BIC (Bayesian information criterion) and the greatest R-square for each antigen. The hypothesis testing of covariates effects was negative to moderately positive effects on the effect of interest (uveitis) in some antigens when type III sum of squares was used for the F-value. The result for the full model was consistent with the selected models in all antigens. This showed that considering all covariates in the model increased the precision of the effect of interest. Correction for multiple testing was done using Benjamini Hochberg false discovery rate in the full model.

Antigens significantly different between patients with and without uveitis were analyzed by hierarchical clustering. Both antigens and samples were analyzed in clustering with “ward.D” and “complete” linkage functions, respectively.

## Results

### Large scale screening methods identified possible JIA autoantigens

**Figure 2:**
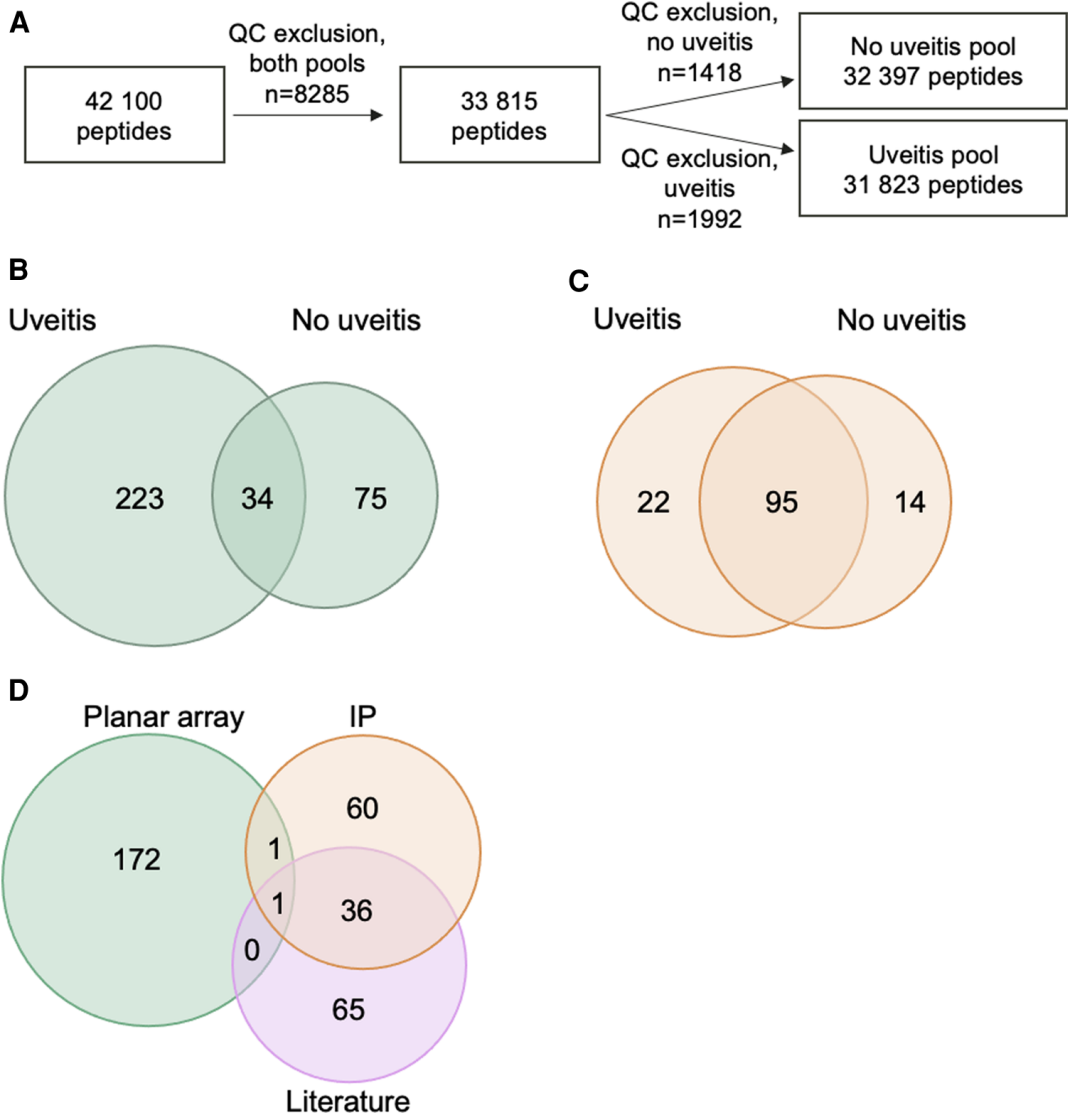
Peptides and proteins detected in large-scale screenings and selection for bead array. (**A**) Flow chart of number of PrEST antigens analyzed on planar array in each pool after quality control (QC) exclusions. (**B**) Venn diagram of PrEST antigen hits in the planar array in the two plasma pools. (**C**) Venn diagram of proteins precipitated by samples from patients in the two plasma pools. (**D**) Venn diagram of the 335 peptides included in bead array, based on source for selection.

More than 31,000 of the PrEST antigens were analyzed in the planar array after quality control exclusions (Figure 2A). Among the analyzed antigens, reactivity was detected towards 332 antigens in at least one of the two plasma pools. Of these, 34 antigens were detected in both pools, 233 antigens were uniquely detected by the uveitis pool, and 75 antigens by the non-uveitis pool (Figure 2B and Supplementary Table S1).

In the immunoprecipitations, a total of 131 proteins were precipitated by at least one of the plasma pools, with an overlap of 95 proteins detected in both patient pools (Figure 2C and Supplementary Table S2).

There was very limited overlap between the two exploratory methods. Only two proteins, fatty acid-binding protein 5 (FABP5) and snRNP-B (SNRPB), were detected by both methods.

To investigate presence of autoantibodies towards identified antigens among JIA patients with and without uveitis, we performed a targeted screening of selected antigens. The targeted array was bead-based and performed on a cohort of 56 patients with oligoarticular- or RF-negative polyarticular JIA (Figure 1C). A total of 339 PrEST antigens were included in the bead array. Four PrEST antigens were excluded from further analysis due to weak coupling to the bead, resulting in a total of 335 peptides in the array. Among the selected PrEST antigens, 174 were chosen from hits in the planar array, 97 from hits in immunoprecipitations, and 102 from proteins identified as autoantigens in JIA or uveitis in the literature (Figure 2D and Supplementary Tables S1–S3). Among the selected PrEST antigens, there was a significant overlap between proteins found by immunoprecipitation and proteins recognized as antigens in the literature (Figure 2D and Supplementary Table S3).

### JIA patients with uveitis have increased reactivity towards several antigens

**Figure 3:**
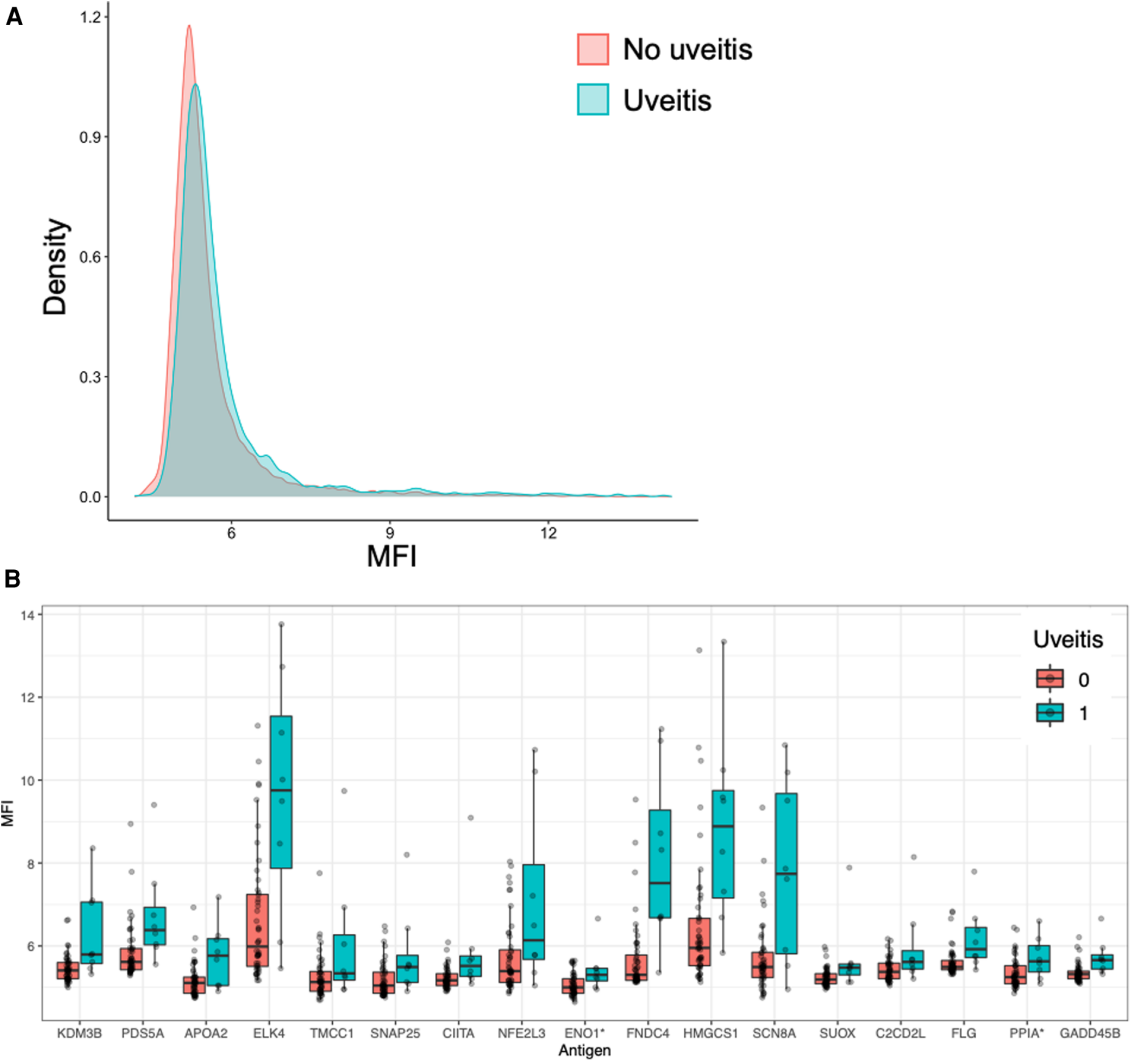
JIA patients with uveitis have increased autoreactivity. (**A**) Histogram of distribution of MFI across all 335 antigens in the bead array, grouped by uveitis. (**B**) Reactivity towards the 17 antigens which were significantly associated with uveitis in the bead array, presented as MFI values. Each dot represents a patient.

To investigate if there were any general difference between reactivity pattern between JIA patients with and without uveitis, we studied the distribution of MFI across all 335 antigens in the bead array. This analysis demonstrated that MFI values in patients with uveitis were higher than the MFI values in patients without uveitis (Figure 3A), in line with the clinical observation that uveitis is associated with presence of autoantibodies.

We then performed a pairwise analysis of reactivity towards all antigens to explore if any specific antigens were associated with uveitis. Individually, reactivity towards 44 antigens was significantly different between patients with and without uveitis, of which 43 were higher and one lower in patients with uveitis compared to patients without uveitis (Supplementary Table S4).

To further evaluate what antigen are relevant we corrected for multiple comparisons and adjusted for covariance with age, IgG, and His6ABP. After this modeling reactivity towards 17 of the antigens remained significantly different, all of them higher in patients with uveitis (Figure 3B and Table 4, Supplementary Table S5).

**Table 4:** List of antigens significantly different in JIA patients with and without uveitis.

Gene	Protein	*p*-value	Ges
KDM3B	Lysine demethylase 3B	0.0001	0.358
PDS5A	PDS5 cohesin associated factor A	0.0009	0.290
APOA2	Apolipoprotein A2	0.0011	0.280
ELK4	ELK4, ETS transcription factor	0.0015	0.267
TMCC1	transmembrane and coiled-coil domain family 1	0.0032	0.242
SNAP25	synaptosome associated protein 25	0.0043	0.231
CIITA	class II major histocompatibility complex transactivator	0.0066	0.213
NFE2L3	nuclear factor, erythroid 2 like 3	0.0116	0.191
ENO1^a^	enolase 1	0.0278	0.159
FNDC4	fibronectin type III domain containing 4	0.0278	0.159
HMGCS1	3-hydroxy-3-methylglutaryl-CoA synthase 1	0.0278	0.156
SCN8A	sodium voltage-gated channel alpha subunit 8	0.0278	0.161
SUOX	sulfite oxidase	0.0352	0.150
C2CD2L	C2CD2 like	0.0411	0.146
FLG	filaggrin	0.0411	0.145
PPIA^a^	peptidylprolyl isomerase A	0.0411	0.142
GADD45B	growth arrest and DNA damage inducible beta	0.0481	0.140

Ges, generalized eta squared.

^a^ Multiple target, peptide is present in more than one protein.

**Figure 4:**
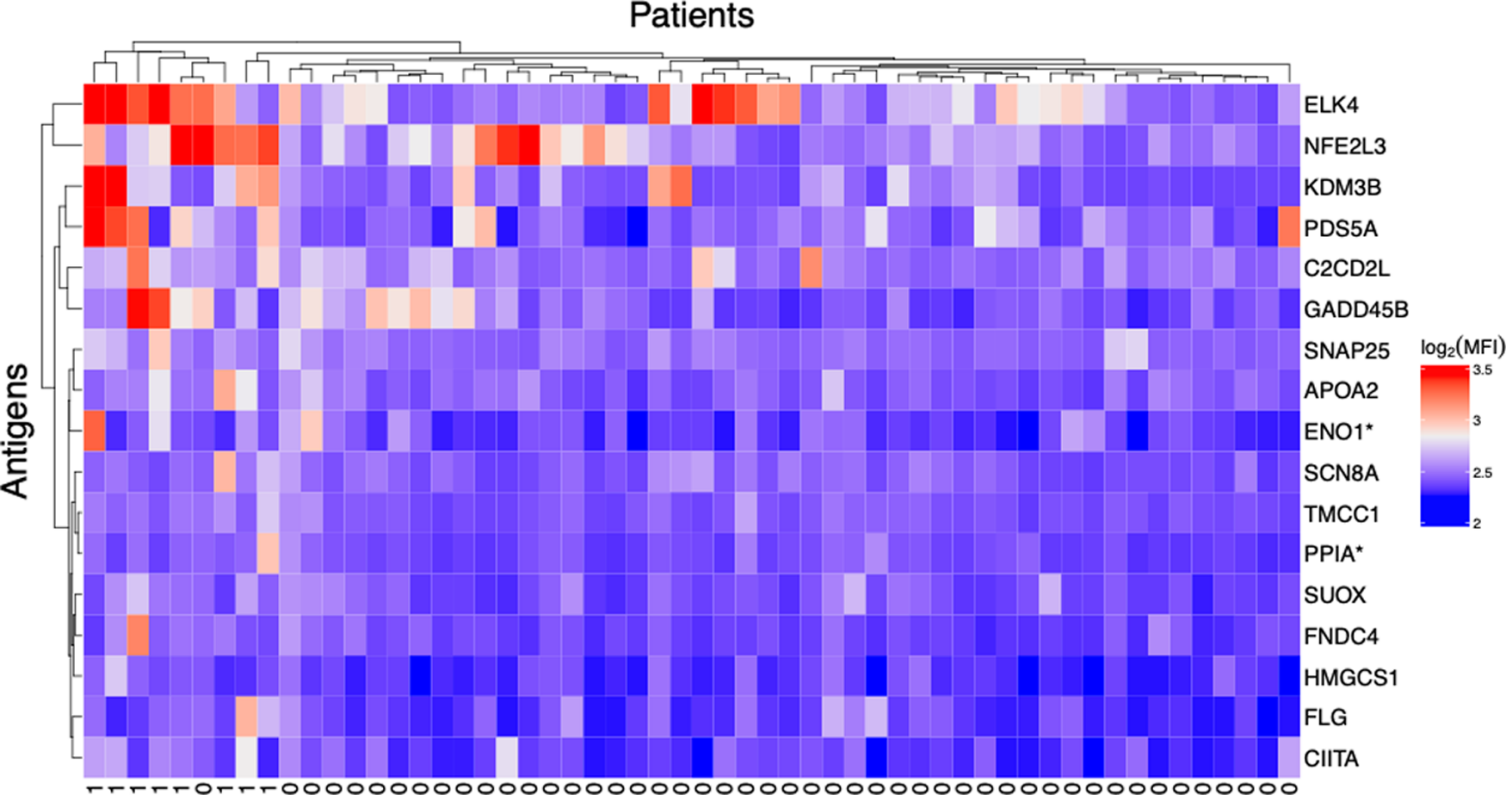
JIA patients with uveitis cluster together based on their autoreactivities. Hierarchical clustering analysis of reactivity towards the 17 peptides significantly different between patients with and without uveitis. Hierarchical clustering is based on MFI distances. Bottom row indicates uveitis (0 = no, 1 = yes).

Next, we performed a hierarchical clustering analysis based on MFI distances on the 17 significantly different antigens (Figure 4). This analysis revealed that patients with uveitis clustered together, with the addition of one patient without uveitis. Despite that reactivity towards all 17 antigens was significantly higher in patients with uveitis, it seems as some antigens are more important in distinguishing between patients with and without uveitis (Figure 4).

## Discussion

In this study, we aimed to identify novel autoantigens in JIA that might be suitable biomarkers for uveitis risk prediction in JIA clinical care. Presence of certain autoantibodies is often used for discrimination between patient subgroups in other fields of rheumatology, but due to the limited knowledge about JIA autoantigens the routinely evaluated antibodies in JIA care are limited to ANA, RF, and antibodies towards cyclic citrullinated peptides (anti-CCP). Identification of specific JIA- and uveitis-associated antigens could provide insight about the immunopathogenesis and greatly improve disease monitoring and diagnostics in JIA. We found that the general autoreactivity was higher in JIA patients with uveitis compared to patients without. Furthermore, we identified that specific autoreactivity towards 17 peptides was significantly increased in patients with uveitis. Cluster analysis showed that the patients with uveitis clustered together, indicating that a combination of certain autoreactivities might be a better predictor for uveitis risk compared to single autoreactivities.

A major strength of our study is the use of both exploratory and targeted analyses. Previous studies on autoantigens in JIA have primarily focused on investigating antigens which are known to be targeted by autoantibodies in other rheumatic and autoimmune diseases, such as histones, ribonucleoproteins, or proteins with post-translational modifications (4, 19, 25–27, 30). To our knowledge, only three studies have used an exploratory approach for identification of novel JIA autoantigens. One of these studies investigated targets for autoantibodies in JIA-related uveitis by analyzing reactivity toward proteins isolated from ocular tissue (22). Another investigated reactivity towards an array of 768 selected proteins (18), and the third used an exploratory phage display approach (31).

To avoid bias and cover as many potential autoantigens as possible, we used two large scale exploratory analyses with completely different methodologies, an autoimmunity profiling planar array and immunoprecipitations from whole cell protein extracts. The planar array includes antigens from approximately 94% of the human proteome, and the antigens in this assay are thus not restricted to expression in a certain cell type, tissue, or developmental stage. Some limitations of the planar array are that the antigens are peptides and not whole proteins, which means that these antigens do not reflect protein secondary structure, folding or post-translational modifications. Additionally, the PrEST antigens are designed to specifically represent certain proteins, and therefore primarily cover areas where a protein is unique and not domains shared between many proteins. In the immunoprecipitations, the protein source was cell lysates with full length proteins having post-translational modifications and three-dimensional structure. However, antigens in the cell lysates are limited to the proteins expressed by the cell line used for the protein extract. Protein abundances within the cells will also affect the likelihood of precipitation and identification. As both methods have obvious strengths and limitations, we believed that the use of both methods would increase the chances to identify relevant JIA autoantigens. To further enhance the likelihood of identifying relevant antigens, we also included peptides from proteins previously recognized as JIA autoantigens in the literature in the targeted array.

The limited overlap between proteins identified by the planar array and immunoprecipitations can most probably be explained by the differences in methodology. Immunoprecipitation was superior to the planar array in detecting proteins which have previously been described as JIA antigens, likely because previous studies have investigated whole proteins in similar settings. The bead array is more similar to the planar array than immunoprecipitations as it uses the same PrEST antigens. However, the bead array is performed in suspension rather than on a surface, which might facilitate antigen secondary structure compared to the planar array. For future studies, knowledge about the limited agreement between antibody analyses of tagged peptides vs. full length proteins is important to consider in the study design.

The JIA patients in this cohort were all of either oligoarticular or RF-negative polyarticular JIA, and the cohort was designed to identify antigens common in these two patient groups which share many clinical features (6, 8). We chose to restrict this study to patients of these subtypes as we believe that there would be too few individuals per group to make appropriate analyses if patients of all seven subtypes would be included. It is known that all individuals, also without autoimmune disease, have a personal autoantibody profile on the peptide arrays which could be considered as a fingerprint or barcode (32). Therefore, a patient cohort with very few individuals per group would risk giving too much weight to individual fingerprints rather than antibody profiles shared among several JIA patients. In our cohort, there were eight individuals with current or a history of uveitis, corresponding to a frequency of 14%. The low number of patients with uveitis represents a limitation to our study, but corresponds with the demographics in the study area (15). In addition, it would have been interesting to compare the autoantibody pattern of the eight children with JIA-related uveitis to children with ANA positive idiopathic uveitis (without arthritis), as they share several similarities. This need to be addressed in future studies.

The results of this study are in line with previous knowledge that JIA-associated uveitis is associated with presence of autoantibodies, especially towards nuclear antigens. In both large-scale methods, we observed a higher number of hits using the uveitis plasma pool compared to the non-uveitis plasma pool, and in the bead array the distribution of MFI values across all 335 antigens was higher in patients with uveitis compared to patients without uveitis. These results suggest that uveitis is associated with a general increase in autoantibodies.

Reactivity towards 17 antigens was significantly higher in patients with uveitis, also after correcting for multiple comparisons and adjustment for covariance. Several of the proteins which were most influential in the clustering analysis, ELK4, NFE2L3, KDM3B, and PDS5A, are nuclear proteins and some are related to DNA processing and handling. Thus, one could speculate that they might contribute to the association between ANA and uveitis.

In the clustering analysis, one patient without uveitis clustered together with the patients with uveitis. We find it likely that some patients in the cohort have a predisposition for uveitis, which is prevented by their treatments. Before the era of biologic treatment options in JIA care, the proportion of patients with uveitis was higher, indicating that the disease-modifying antirheumatic drug- (DMARD) treatment might prevent development of uveitis (15, 33). This phenomenon might explain how some patients without uveitis can have a similar autoantibody pattern as patients with uveitis. JIA patients with an autoantibody pattern resembling patients with uveitis might be at high risk of developing uveitis, especially if untreated.

As autoantibody reactivities were evaluated towards PrEST antigens and not proteins in the bead array, it is difficult to conclude if antibodies will bind the full-length folded proteins in a physiological setting. However, reactivity towards the PrEST antigens identified to be associated with uveitis in this study could be interesting as biomarkers for uveitis prediction in JIA.

In conclusion, this study used a novel approach for identification of autoantigens in JIA and uveitis. We combined two exploratory methods investigating antibodies towards the whole human proteome, and one targeted array of selected peptides, with the aim of discovering autoantibodies which could be used as novel biomarkers for JIA and uveitis prediction. We identified that JIA patients with uveitis had a higher level of autoreactivity in general, and specific autoantibodies towards 17 antigens. We found that the patients with uveitis shared an autoreactivity pattern and clustered together in a hierarchical clustering analysis. This study of autoantibodies associated with uveitis in JIA could be a starting point for identification of prognostic biomarkers useful in JIA clinical care, where these 17 peptides are interesting targets.

## Data Availability

The original contributions presented in the study are included in the article/**Supplementary Material**, further inquiries can be directed to the corresponding author/s.
